# Operational experience of the Dutch helicopter emergency medical services (HEMS) during the initial phase of the COVID-19 pandemic: jeopardy on the prehospital care system?

**DOI:** 10.1007/s00068-020-01569-w

**Published:** 2021-01-12

**Authors:** Quinten G. H. Rikken, Sarah Mikdad, Mathijs T. Carvalho Mota, Marcel A. De Leeuw, Patrick Schober, Lothar A. Schwarte, Georgios F. Giannakopoulos

**Affiliations:** 1 Trauma Center, Department of Surgery, Location AMC and Location VUmc, Amsterdam University Medical Centres, Meibergdreef 9, 1105 AZ Amsterdam, The Netherlands; 2 Department of Anesthesiology, Location AMC and Location VUmc, Amsterdam University Medical Centres, Amsterdam, Netherlands

**Keywords:** Helicopter emergency medical services, Trauma, COVID-19, SARS-CoV2

## Abstract

**Purpose:**

The SARS-CoV-2 virus has disrupted global and local medical supply chains. To combat the spread of the virus and prevent an uncontrolled outbreak with limited resources, national lockdown protocols have taken effect in the Netherlands since March 13^th^, 2020. The aim of this study was to describe the incidence, type and characteristics of HEMS and HEMS-ambulance ‘Lifeliner 1’ dispatches during the initial phase of the COVID-19 pandemic compared to the same period one year prior.

**Methods:**

A retrospective review of all HEMS and HEMS-ambulance ‘Lifeliner 1’ dispatches was performed from the start of Dutch nationwide lockdown orders from March 13th until May 13th, 2020 and the corresponding period one year prior. Dispatch-, operational-, patient-, injury-, and on-site treatment characteristics were extracted for analysis. In addition, the rate of COVID-19 positively tested HEMS personnel and the time physicians were unable to take call was described.

**Results:**

During the initial phase of the COVID-19 pandemic, the HEMS and HEMS-ambulance was requested in 528 cases. One year prior, a total of 620 requests were received. The HEMS (helicopter and ambulance) was cancelled after deployment in 56.4% of the COVID-19 cohort and 50.7% of the historical cohort (*P* = 0.05). Incident location type did not differ between the two cohorts, specifically, there was no significant difference in the number of injuries that occurred at home in pandemic versus non-pandemic circumstances. Besides a decrease in the number of falls, the distribution of mechanisms of injury remained similar during the COVID-19 study period. There was no difference in self-inflicted injuries observed. Prehospital interventions remained similar during the COVID-19 pandemic compared to one year prior. Specifically, prehospital intubation did not differ between the two cohorts. The rate of COVID-19 positively tested HEMS personnel was 23.1%. Physicians who tested positive were unable to take call for a mean of 25 days (range 8–53).

**Conclusion:**

A decrease in the number of deployments and increase in the number of cancelled missions was observed during the COVID-19 study period. No major differences in operational- and injury characteristics were found for HEMS and HEMS-ambulance dispatches between the initial phase of the COVID-19 pandemic in the Netherlands and the same period one year prior. These findings highlight the importance of continued operability of the HEMS, even during pandemic circumstances.

**Level of evidence:**

III, retrospective comparative study.

## Introduction

Treatment of severely injured patients requires rapid medical intervention. In the Netherlands, the Helicopter Emergency Medical Services (HEMS) and HEMS-ambulance are dispatched to treat severely injured patients [1, 2]. Up to 7600 deployments occur annually among four HEMS regions. The purpose of HEMS deployment is to rapidly transport the specialized team to the scene and consequently to add opportunity for advanced prehospital procedures, such as advanced airway management, chest tube placement, medication administration and surgical interventions [3]. As such, HEMS forms an integral part of the prehospital emergency medical care chain.

The SARS-CoV-2 virus has disrupted global and local medical supply chains [4–6]. Although many non-emergency medical and surgical conditions can be treated after the rapid escalation of COVID-19 infections has stabilized, emergency conditions and trauma cases still require immediate assessment and timely resolution. To combat the spread of the virus and prevent an uncontrolled outbreak with limited resources, national lockdown protocols have taken effect in the Netherlands since March 13th 2020 [7]. As a result, the incidence of trauma-related injuries are thought to decrease during the national lockdown. It is hypothesized that this decrease may be attributable to the decrease in the trauma population at risk. However, evidence exists on the negative effects of social isolation on physical- and mental health during the COVID-19 pandemic, which could result in a shift towards more dispatches for domestic violence, excessive drug abuse and suicides [8].

Currently, the indications and principles for the management of the acutely injured have remained similar as in non-pandemic circumstances. However, it remains to be elucidated whether the incidence and type of medical emergency responses during a nationwide lockdown have altered compared to a non-lockdown period. Therefore, the present study aims to describe the HEMS (including HEMS-ambulance) operations during the COVID-19 pandemic and compare these to the same time period one year prior. In addition, the authors describe their initial experience with HEMS safety and precaution protocols during the pandemic period and the rate of COVID-19 infections among HEMS personnel.

## Methods

### Dispatch and patient selection

**Fig. 1:**
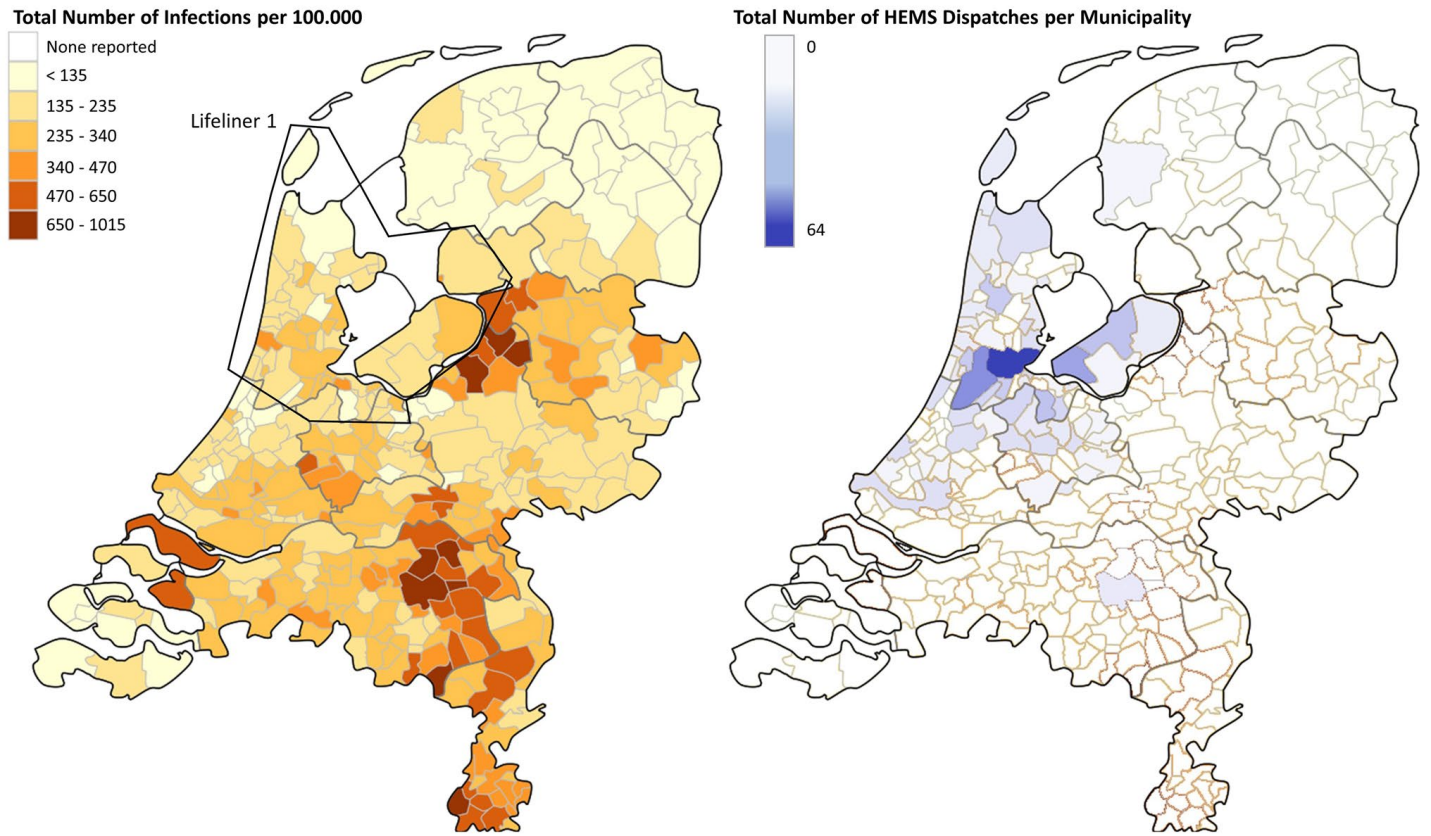
Geographical representation per municipality of left) the total number of COVID-19 infections per 100.000 during the studied period and HEMS Lifeliner 1 operational area, and right) the total number of HEMS Lifeliner 1 dispatches from the operational area. Source: Rijksinstituut voor Volksgezondheid en Milieu (RIVM), Epidemiologische situatie COVID-19 in Nederland, May 13th 2020

The Dutch HEMS are distributed among four operational areas in the Netherlands (Fig. 1), and provide rapid emergency physician-based medical services to severely injured patients or critically ill patient as they deliver a wide spectrum of advanced (trauma) care at the scene [9]. The HEMS and HEMS-ambulance are able to reach up to 80% of the Dutch population within 15 min, of which the ‘Lifeliner 1’, operating in the Region North-West Netherlands and stationed in Amsterdam, covers 2.7 million inhabitants [3, 10]. The HEMS are dispatched in addition to standard ambulance care, are operational 24/7, and can also be transported when needed by a designated road ambulance vehicle (HEMS-ambulance) in case of logistical, technical or meteorological obstacles [11]. When a patient is ready for ambulance transport and the HEMS or HEMS-ambulance have not yet arrived at the scene, the ambulance and HEMS will commonly *rendezvous* en route to transport the patient to the hospital [11]. Data are registered by physicians and assistants post-operation and cross-checked for completeness.

The operational database of the HEMS Lifeliner 1 was reviewed for all dispatches over a 3 month period, starting at the introduction of the nationwide COVID-19 restrictions (March 13th until May 13th 2020) and the same period one year prior. All identifiable dispatches and dispatch cancellations were included.

### Data collection

Data regarding the following operational characteristics were extracted: incident location, dispatch time (at injury site and to hospital), HEMS mode of transport (ambulance vehicle or helicopter), primary call (dispatched simultaneously with paramedic based EMS) or secondary call (requested by EMS on site), cancelation rate and -reason, the use of secondary transport from landing to injury site, rendezvous rate, and the rate of patient air transportation.

Patient- and injury characteristics included were: patient age category, gender, number of patients involved, cause-, mechanism- and intention of injury, incident location, and the National Advisory Committee for Aeronautics (NACA) score [12]. The NACA score is a score for injury severity that ranges from zero (no injury or disease) to seven (death) and is used to determine the level of required medical attention.

On-site treatment characteristics included were: on site emergency interventions (e.g., advanced airway management), on site cardiopulmonary resuscitation (CPR), CPR termination rate, and the prehospital mortality rate. In case of missing data, data was labeled as ‘unknown’.

### Safety protocol

Protocols to ensure health and safety of the HEMS team were implemented since the first recorded case of COVID-19 in the Netherlands (February 27th 2020). During the working shifts, HEMS crewmembers were keeping 1.5 m distance as much as possible and performing frequent hand hygiene. HEMS staff conducted regular health screenings, including symptom screening, before starting their shift. During the operations, both HEMS physician and nurse were wearing surgical masks and protective eyewear. In case of advanced airway management, the physician was wearing a complete protective package (protective gown, gloves, filtering face piece [FFP]-2 mask, protective eyewear and hat) in addition to the standard issued long-sleeve shirts/overalls. It was agreed to not transport patients by helicopter when there was no medical reason to do so to limit contact of HEMS personnel with patients. After the operation, all protective materials were disposed of properly or cleaned according to protocol. Both helicopter and ambulance vehicle were equipped with enough protective packages for several missions. HEMS staff members quarantined and underwent immediate testing when symptoms compatible with COVID-19 were present (Table 1).

**Table 1:** Patient- and dispatch characteristics from the COVID-19 and historical cohort

	COVID-19 group (*n* = 230) From March 13th to May 13th 2020	Control group (*n* = 306) From March 13th to May 13th 2020	Total (*n* = 536)	*P* value
Age category, *n* (%)				0.292
Adult	187 (81.3%)	260 (85.0%)	447 (83.4%)	
Pediatric	40 (17.4%)	37 (12.0%)	77 (14.4%)	
Adult and pediatric	1 (0.4%)	3 (1.0)	4 (0.7%)	
Unknown	2 (0.9%)	6 (2.0%)	8 (1.5%)	
Male, *n* (%)	114 (49.6%)	145 (47.4%)	259 (48.3%)	0.663
Mode of transport, *n* (%)				** < 0.001**
Ambulance vehicle	59 (25.7%)	122 (39.9%)	180 (33.6%)	
Helicopter	171 (74.3%)	184 (60.1%)	354 (66.0%)	
Reason for transport via HEMS- ambulance vehicle, *n* (%)				
Distance/time	55 (23.9%)	109 (35.6%)	164 (30.6%)	0.515
Weather	3 (1.3%)	5 (1.6%)	8 (1.5%)	
Technical	0 (0.0%)	4 (1.3%)	4 (0.7%)	
Other	1 (0.4%)	1 (0.3%)	2 (0.4%)	
Helicopter used for patient transport, *n* (%)	0 (0.0%)	7 (2.3%)	7 (1.3%)	**0.032**
Transport Time (mins), Median (IQR)	10 [7–14]	10 [7–15]	10 [7–14]	0.679
NACA (0–7), Median (IQR)	4 [3–6]	4 [3–5]	4 [3–5]	0.147
Incident location type, *n* (%)				
Home	50 (21.7%)	40 (13.1%)	90 (16.8%)	0.178
Road	74 (32.2%)	97 (31.7%)	171 (31.9%)	
Workplace	10 (4.3%)	11 (5.9%)	21 (3.9%)	
Sport facility	0 (0.0%)	2 (1.1%)	2 (0.4%)	
Other	6 (2.6%)	15 (4.9%)	21 (3.9%)	
Unknown	90 (39.1%)	141 (46.1%)	231 (43.1%)	
Rendezvous, *n* (%)	14 (6.1%)	32 (10.5%)	46 (8.6%)	0.087
Time of dispatch, *n* (%)				
Day	172 (74.8%)	218 (71.2%)	390 (72.8%)	0.379
Night	58 (25.2%)	88 (28.8%)	146 (27.2%)	
Type of HEMS physician, *n* (%)				
Trauma surgeon	42 (18.3%)	32 (10.5%)	74 (13.8%)	**0.011**
Anesthesiologist	188 (81.7%)	274 (89.5%)	462 (86.2%)	
Type of HEMS operation, *n* (%)				
Primary	201 (87.4%)	255 (83.3%)	456 (85.1%)	0.221
Secondary	29 (12.6%)	51 (16.7%)	80 (14.9%)	
Type of dispatch, *n* (%)				0.928
Trauma	143 (62.2%)	191 (62.4%)	334 (62.3%)	
Non-trauma	87 (37.8%)	115 (37.6%)	202 (37.7%)	
Cause of injury, *n* (%)				
Traffic accident	64 (27.8%)	74 (24.2%)	138 (25.7%)	> 0.99
Fall	45 (19.6%)	76 (24.8%)	121 (22.6%)	**0.022**
Stab wound	19 (8.3%)	21 (6.9%)	40 (7.5%)	> 0.99
GSW	6 (2.6%)	7 (2.3%)	13 (2.4%)	> 0.99
Other	9 (3.9%)	13 (4.2%)	22 (4.1%)	> 0.99
Mechanism of injury, *n* (%)				
Blunt	115 (50.0%)	150 (49.0%)	265 (49.4%)	0.422
Penetrating	24 (10.4%)	27 (8.8%)	51 (9.5%)	> 0.99
Mixed	4 (1.7%)	14 (4.6%)	18 (3.4%)	**0.031**
Intention of injury, *n* (%)				
Assault	15 (6.5%)	27 (8.8%)	42 (7.8%)	0.142
Self-inflicted	25 (10.9%)	35 (11.4%)	60 (11.2%)	0.262
Prehospital interventions, *n* (%)				
Advanced airway management	64 (27.8%)	77 (25.2%)	141 (25.3%)	0.490
IV access	96 (41.7%)	124 (40.5%)	220 (41.0%)	0.791
Pressure bandage	3 (1.3%)	9 (2.9%)	12 (2.2%)	0.249
Tourniquet	2 (1.2%)	4 (2.2%)	6 (1.7%)	0.686
Immobilization	5 (2.2%)	16 (5.2%)	21 (3.9%)	0.076
Pelvic stabilization	16 (9.4%)	18 (9.8%)	34 (9.6%)	> 0.99
Thoracotomy	1 (0.6%)	0 (0.0%)	1 (0.3%)	0.480
Needle thoracostomy	7 (4.1%)	5 (2.7%)	12 (3.4%)	0.563
Surgical thoracostomy	7 (%)	9 (%)	16 (%)	0.789
Prehospital mortality, *n* (%)	14 (6.1%)	19 (6.2%)	33 (6.2%)	> 0.99
CPR, *n* (%)	39 (17.0%)	35 (11.4%)	74 (13.8%)	0.077
Termination of CPR resuscitation**,** *n* (%)	12 (5.2%)	13 (4.2%)	25 (4.7%)	0.842

Significant differences are in bold

*IQR* interquartile range; *MMT* mobile medical team; *HEMS* helicopter emergency medical services; *NACA* National Advisory Committee on Aeronautics; *GSW* gunshot wound; *IV* intravenous; *CPR* cardiopulmonary resuscitation

### Statistical analysis

Operational- and patient characteristics were summarized using descriptive statistics with absolute numbers and percentages for categorical variables, and means with standard deviations for continuous variables. Data was visually assessed for normality using histograms and boxplots, and with a Shapiro–Wilk test. Dispatch and operational characteristics between the COVID-19 and the historical control group were compared using Fisher’s Exact test for dichotomous outcomes and two-sided ANOVA for continuous variables. A sub-analysis was performed to evaluate the dispatch- and cancellation rates over time using a Fisher’s Exact test. The rate of COVID-19 positively tested HEMS personnel was calculated by dividing the number of positively tested staff by the total number of HEMS staff. A two-sided level of *P* < 0.05 was considered significant. Data analysis was performed using Stata 15 (StataCorp LP, College Station, TX).

## Results

### Dispatch characteristics

**Fig. 2:**
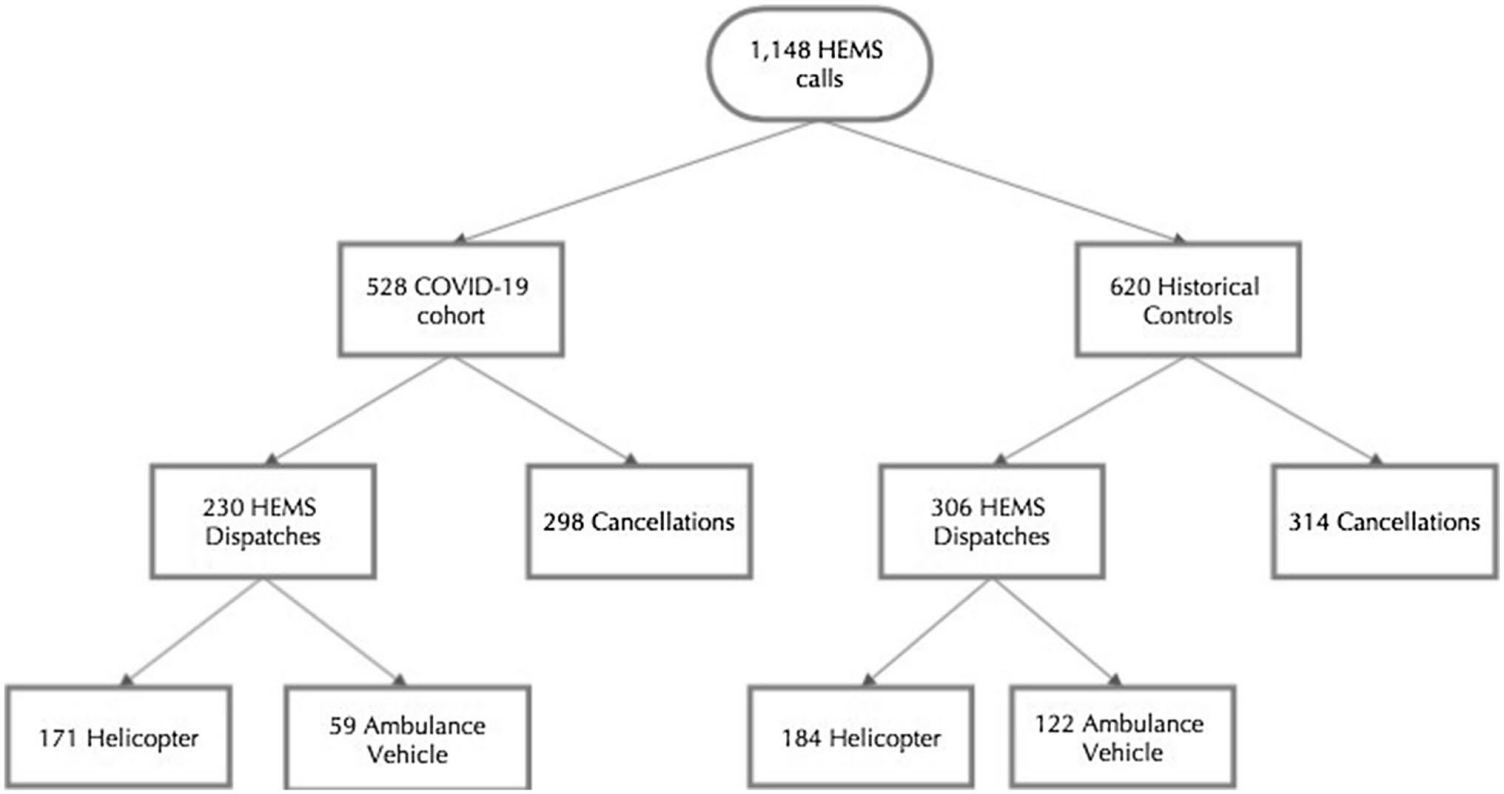
Flowchart of dispatch selection

During the COVID-19 pandemic in the Netherlands, the HEMS Lifeliner 1 was requested in 528 cases (Fig. 2). This corresponds to a reduction by 14.8% compared to the same time period in the previous year, in which a total of 620 requests were received. A geographical overview of COVID-19 infections in the Netherlands and HEMS dispatches per municipality is shown in Fig. 1.

### Cancellation characteristics

HEMS was cancelled after deployment in 56.4% of the COVID-19 cohort and 50.7% in the historical cohort (Table 2). Of all cancellations during the COVID-19 pandemic, 42.1% did not have a medical indication for advanced medical care compared to 38.9% in the historical cohort. No difference was found in time to cancellation between the two cohorts.

**Table 2:** HEMS cancellations from COVID-19 and historical cohort

	COVID-19 group (*n* = 528)	Control group (*n* = 620)	Total (*n* = 1148)	*P* value
HEMS cancelled, *n* (%)	298 (56.4%)	314 (50.7%)	612 (53.3%)	0.050
HEMS cancel reason, *n* (%)				0.030
No advanced medical indication	222 (42.1%)	241 (38.9%)	463 (40.3%)	
Patient declared dead	28 (5.3%)	24 (3.9%)	52 (4.5%)	
HEMS transport time to patient too long	22 (4.2%)	9 (1.5%)	31 (2.7%)	
HEMS transport not possible (technical, weather)	2 (0.4%)	1 (0.2%)	3 (0.3%)	
Unknown	19 (3.6%)	25 (4.0%)	44 (3.8%)	
Time to cancelation (mins), median (IQR)	7 [6–10]	7 [5–10]	7 [6–10]	0.538

*IQR* interquartile range

### Dispatch and cancellation over time

**Fig. 3:**
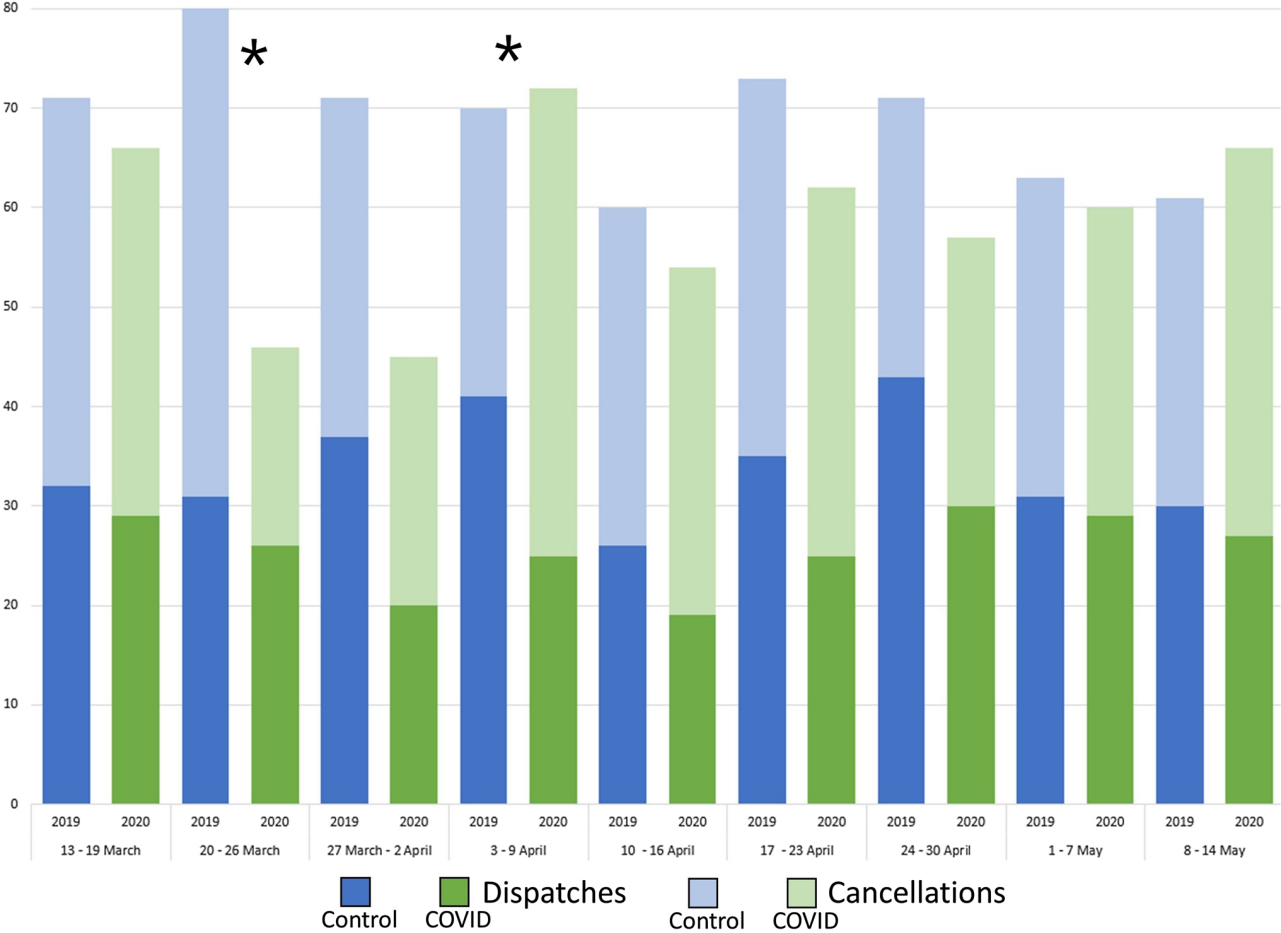
Number of HEMS-calls and cancelled missions during the COVID-19 pandemic compared to 1 year prior. *Significant difference between the number of deployments and cancellations between the COVID-19 study period and the control study period for a given week

During the COVID-19 pandemic the number of HEMS dispatches differed over time (Table 2). Specifically, the number of dispatches and cancelled missions in week 2 (20–26 March) and week 4 (3–9 April) were significantly less frequent in the COVID-19 study period compared to the same weeks 1 year prior (Fig. 3). The mean time from dispatch to the scene was 10 min for both pandemic and non-pandemic circumstances.

### Operational characteristics

An overview of the operational characteristics is shown in Table 1. There were significantly more HEMS than HEMS-ambulance vehicle dispatches during the COVID-19 pandemic compared to one year prior, with as foremost reason distance/time for patient transport. The HEMS was not used to transfer patients from the scene to the hospital during the COVID-19 pandemic, whereas in the historical cohort it was used in seven missions. There was no difference in trauma-related dispatch rate between the two cohorts. Incident location type did not differ between the two cohorts, specifically, there was no difference in the number of injuries that occurred at home in pandemic versus non-pandemic circumstances. The number of falls significantly decreased during the COVID-19 pandemic. There was no difference in self-inflicted injuries observed.

No differences in prehospital interventions were observed during the COVID-19 pandemic compared to one year prior. Specifically, advanced airway management did not differ between the two cohorts. Resuscitative efforts were not significantly different between the two study periods and accounted for 24.1% of the COVID-19 cohort and 17.2% of the historical cohort. Return of spontaneous circulation could not be achieved in 5.2% of the COVID-19 cohort and 4.2% in the historical cohort, respectively.

### Severity of medical emergencies and prehospital mortality

The median NACA score was 4.0 in both groups. Correspondingly, the prehospital mortality rate observed was 6.1% in the pandemic compared to 6.2% in the non-pandemic cohort.

### Infections among HEMS staff

The number of symptomatic, positively tested HEMS personnel during the study period was three out of 13 (23.1%). The physicians who tested positive were unable to take call for a mean of 25 days (range 8–53).

## Discussion

This is the first study to describe the experiences of a physician staffed HEMS during the COVID-19 pandemic and to compare these data to non-pandemic circumstances. No differences in operational- and medical characteristics were found for HEMS and HEMS-ambulance dispatches between the initial phase of the COVID-19 pandemic in the Netherlands and the same time frame 1 year prior.

Overall, a decrease in incidence of mission dispatches (i.e., non-canceled dispatches) was observed during the COVID-19 pandemic compared to the historical cohort. This decrease in dispatches and increase in cancelled missions was primarily observed in the first month of the COVID-19 pandemic in the Netherlands. Ambulance vehicle dispatches were less frequent than 1 year prior, whereas helicopter dispatches gained in operability during the COVID-19 pandemic. The foremost reason for deployment by HEMS-ambulance vehicle was distance and time, rather than meteorological conditions compared to the prior year.

In terms of the cause of injury, this study found a significant decrease in the number of falls during the COVID-19 study period. This could be due to a lower number of work-related and intoxication-related accidents. However, we were not able to obtain detailed injury cause data due to the retrospective design of the study and the nature of data collection. No increase in violence related injuries (i.e., gunshot wounds (GSW) and stabbings) was observed. In the United States reports from increased violence related—and penetrating injuries (primarily from GSW) have emerged, posing a challenge for the, arguably, already strained health system [13]. In the Netherlands, however, a relatively small proportion of the total number of trauma patients has penetrating injuries in both non-pandemic [14] and pandemic circumstances.

Previous experience from the influenza pandemic and SARS epidemic has shown how mental health can be adversely affected and how it can potentially lead to an increase in suicide ideation and behavior in the population at risk [15]. In this study, the rate of self-inflicted injuries did not significantly differ from the historical control group. However, as the present study is limited to 3 months and solely includes HEMS dispatch data the long-term effects of prolonged social isolation and -distancing on the incidence of self-inflicted injuries remain to be elucidated [16]. Therefore, future efforts should focus on closely monitoring underlying mental health related injuries during the current pandemic.

As prehospital care providers operate along the frontline, they are considered the foundation of maturated trauma systems. Due to the time sensitive nature of the emergency dispatches and inability to screen patients for COVID-19 before treatment, HEMS potentially have a relatively high exposure rate to COVID-19. Safety procedures and protective measures are, therefore, crucial for HEMS and emergency medical staff. The safety protocol used in the present study can be considered similar to those used by other European HEMS services, as described by Hilbert-Carius et al. [17]. These guidelines can be used to ensure safe and sustained operability of emergency medical services. The potential loss of prehospital care providers as a result of becoming infected can be considered a serious threat to the prehospital care chain. In the Netherlands, HEMS physicians consist of a team of trauma surgeons or anesthesiologists. At our institution, three HEMS physicians were unable to take call for a mean 25 (range 8–53) days due to positive testing for COVID-19. This was primarily due to the prolonged absence of one physician, who did not return to the HEMS service in the study period. Moreover, one HEMS physician had obligations to take additional in-hospital tasks related to COVID-19, causing a gap in operational capacity within the team [18]. Trauma surgeons, therefore, took significantly more calls during the COVID-19 pandemic compared to 1 year prior to remain operable. Even though at the start of the COVID-19 outbreak in the Netherlands a rigorous personal protective protocol was implemented, care provider infections were imminent and posed a substantial stress on the operability of the HEMS.

As rapid transport is pivotal to decrease prehospital mortality the question arises whether prehospital care systems are being overwhelmed by COVID-19 patients and are unable to maintain the same level of responsiveness for severely ill or injured patients. No difference was observed between the time that the call was made to dispatch the HEMS and the arrival on the scene. Compatible with this finding, the rate of prehospital fatalities remained similar as in non-pandemic circumstances.

In terms of operability, the HEMS performed roughly a similar number of prehospital interventions during the COVID-19 pandemic compared to 1 year prior. Specifically, similar rates of advanced airway management were observed. A study by Tran et al. evaluated the risk of transmission of SARS-CoV-1 to health care providers performing endotracheal intubation and found a 6.6 higher risk of infection [19]. As the tracheal viral load is considered to be high in COVID-19 patients, prehospital endotracheal intubation can also be considered a high-risk intervention for the exposure to SARS-CoV-2, showing the need for personal protective equipment in prehospital care personnel [20].

In the beginning of April 2020 the American College of Surgeons (ACS) stated concerns with regards to trauma centers, fearing operability could potentially be compromised due to the surging number of COVID-19 cases in the US [21]. In the state of New York, novel CPR protocols have emerged to achieve early prehospital triage that meets the trauma center capacity. Cardiopulmonary resuscitation in these patients is currently withheld when the return of spontaneous circulation (ROSC) is not achieved [22]. In this study, we did not observe differences in initial resuscitation efforts performed on the scene by HEMS, nor differences in termination of resuscitation when ROSC could not be achieved.

The findings of the present study have to be interpreted within the scope of its design. The retrospective observational design reduces the strength of the conclusions from the statistical analysis. Furthermore, this study is limited to the operations of the HEMS Lifeliner 1, in one of the four Dutch HEMS operational areas. Therefore, the national distribution during the COVID-19 pandemic is not included in this study. As the scope of this study was primarily to describe prehospital characteristics associated with the operability of HEMS during the COVID-19 pandemic, the present study did not include in-hospital outcomes of the studied patient sample. Currently, a multicenter study at our institution is ongoing to capture in-hospital outcomes of both trauma- and non-trauma related presentations at the emergency room and to monitor the incidence of underlying mental-health related injuries during the COVID-19 pandemic.

## Conclusion

The reported incidence of HEMS deployments decreased and the number of cancelled missions increased in the initial phase of the COVID-19 pandemic in the Netherlands compared to the same period 1 year prior. No major differences in operational- and injury characteristics were found for HEMS and HEMS-ambulance dispatches in both study periods. Safety protocols are crucial to limit the risk of COVID-19 cases among HEMS personnel, as the continued operability of HEMS during pandemic circumstances is pivotal to the prehospital emergency care chain. Future efforts that monitor in-hospital outcomes of both trauma- and non-trauma related injuries during pandemic circumstances are needed.
